# Planar Wide-Angle Imaging System with a Single-Layer SiC Metalens

**DOI:** 10.3390/nano15131046

**Published:** 2025-07-05

**Authors:** Yiyang Liu, Qiangbo Zhang, Changwei Zhang, Mengguang Wang, Zhenrong Zheng

**Affiliations:** College of Optical Science and Engineering, Zhejiang University, Hangzhou 310027, China

**Keywords:** metasurfaces, metalens, wide field of view, imaging

## Abstract

Optical systems with wide field-of-view (FOV) imaging capabilities are crucial for applications ranging from biomedical diagnostics to remote sensing, yet conventional wide-angle optics face integration challenges in compact platforms. Here, we present the design and experimental demonstration of a single-layer silicon carbide (SiC) metalens achieving a 90° total FOV, whose planar structure and small footprint address the challenges. This design is driven by a gradient-based numerical optimization strategy, Gradient-Optimized Phase Profile Shaping (GOPP), which optimizes the phase profile to accommodate the angle-dependent requirements. Combined with a front aperture, the GOPP-generated phase profile enables off-axis aberration control within a planar structure. Operating at 803 nm with a focal length of 1 mm (NA = 0.25), the fabricated metalens demonstrated focusing capabilities across the wide FOV, enabling effective wide-angle imaging. This work demonstrates the feasibility of using numerical optimization to realize single-layer metalens with challenging wide FOV capabilities, offering a promising route towards highly compact imagers for applications such as endoscopy and dermoscopy.

## 1. Introduction

Wide field-of-view (FOV) imaging systems, enabling the capture of extensive information within a single exposure, are increasingly demanded across diverse fields [1]. However, conventional wide-angle lenses are typically designed with complex, cascaded assemblies of multiple refractive elements to mitigate off-axis aberrations [2]. This approach results in bulky form factors, intricate fabrication, and significant challenges for integration into miniaturized platforms, conflicting with modern technological trends.

Metasurfaces have emerged as a compelling alternative, offering a new paradigm for planar optics. By engineering subwavelength nanostructures on a flat substrate, metasurfaces allow unprecedented control over the phase, amplitude, and polarization of light within an ultrathin profile [3,4,5,6,7,8,9]. This capability enables the functional compression of bulky optical systems and holds particular promise for imaging applications demanding compactness and precision. Building on these advances, metasurfaces have been extended to a wide range of practical implementations, such as planar lenses [10,11,12,13], holographic displays [14,15,16], vortex beam generators [17], and polarization modulators [18,19]. Specifically, various strategies have explored the potential of metasurfaces for wide-angle imaging, where angle-dependent off-axis aberrations pose a critical challenge. Early theoretical work analyzed aberrations under oblique incidence, leading to the design of an aplanatic metasurface on curved substrates for aberration correction [20]. While theoretically effective, curved configurations pose significant fabrication challenges and limit integrability. To address this, dual-layer planar metasurfaces inspired by compound lenses have been developed, demonstrating enhanced field-of-view (FOV) performance while simplifying fabrication [21,22]. Furthermore, subsequent developments replaced the first metasurface of the doublet with a small physical aperture to enhance off-axis performance while reducing structural complexity [23,24]. Concurrently, various optimization techniques have been applied on the computational design front, ranging from optimized sinusoidal phase profiles [25] to epsilon-greedy algorithms that tune the width and position of concentric nanorings in metalens [26]. More recently, fueled by advances in computational imaging and machine learning, researchers have explored wide-FOV image stitching from double polarization channels or multiple angular channels captured via metasurfaces [27,28]. Additionally, deep learning approaches have been applied to reconstruct images from optics for better wide-FOV performance [29,30], and even end-to-end joint optimization using neural networks for enhanced angular coverage has been pursued [31].

Despite substantial progress in extending the field-of-view (FOV) capabilities of metasurfaces, many existing designs still depend on multilayer architectures or intricate optimization pipelines, which can limit their scalability and integrability in practical applications. Achieving a balance between off-axis aberration correction, structural simplicity, and fabrication feasibility remains an open issue when targeting ultra-wide-FOV. In this work, we propose a single-layer metasurface that achieves a 90° FOV. This capability is realized through a dedicated strategy, which integrates a gradient-based numerical optimization of the metasurface phase profile (Gradient-Optimized Phase Profile Shaping—GOPP) along with a decoupled front aperture serving as a basic means of angular control. The resulting device, fabricated for near-infrared operation at *λ* = 803 nm, features an effective aperture diameter of 0.5 mm and a numerical aperture (NA) of 0.25. Prior studies have demonstrated that multilayer metalenses usually offer relatively high numerical apertures and broader angular coverage—such as an F number of 0.9 with a 60° FOV [21], and 0.44 NA with a 50° FOV [22]. While single-layer implementations tend to reduce fabrication complexity and provide moderate FOVs, with reported FOVs ranging from 30° to 40° at near-infrared and visible wavelengths [23,26]. Building upon these efforts, our design delivers a relatively wider FOV while maintaining the inherent simplicity and compactness of a single-layer structure, providing a viable pathway toward miniaturized optical systems with broad angular coverage.

## 2. Methods

**Figure 1:**
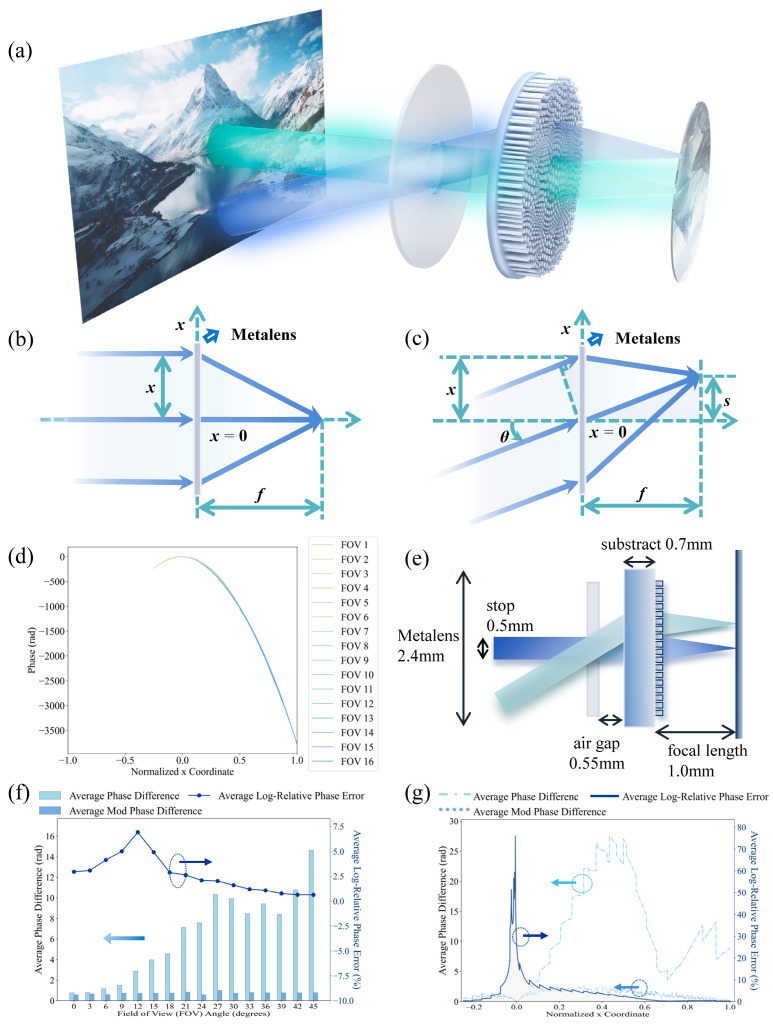
Wide-FOV metalens design and analysis. (**a**) A conceptual illustration of the metasurface imaging system. (**b**) The focusing control for normal incidence beams by the hyperbolic metalens. (**c**) The focusing control for oblique incidence beams at a specified angle by metalens. (**d**) Target phase profile along the metasurface radius after compensation. (**e**) An illustration of the structural parameters of the designed metasurface. (**f**) The average phase difference metrics with respect to different incident angles. (**g**) The average phase difference metrics with respect to different axial coordinates.

This configuration, conceptually illustrated in Figure 1a, leverages the ability of the metasurface to shape the wavefront for focusing and aberration control, while the front aperture serves to constrain the angular distribution of incident light.

Achieving consistent imaging performance across a wide-FOV with a single metasurface element is nontrivial. For ideal on-axis focusing, a simple hyperbolic phase profile is sufficient to convert a plane wavefront into a spherical wavefront converging to a single focal point. The required phase profile Δ*ϕ* at a position (*x*, *y*) is given by the following [32,33]:

(1) $$\Delta \phi =-\frac{2\pi }{\lambda }\left(\sqrt{x^{2}+y^{2}+f^{2}}-f\right),$$

where *λ* is the wavelength and *f* is the focal length.

Unlike the ideal on-axis focusing scenario illustrated schematically in Figure 1b, the optimal phase distribution required for wide-angle aberration correction is strongly angle-dependent. To define the target for our design, we first consider the ideal phase profile required to focus light incident at a specific angle *θ* to its corresponding image point on the focal plane. As illustrated in Figure 1c, this ideal phase profile can be expressed as Equation (2) [34]:

(2) $$\Delta \phi =-\frac{2\pi }{\lambda }\left[\sqrt{{\left(x-s\right)}^{2}+f^{2}}-\sqrt{s^{2}+f^{2}}+x\sin\theta \right],$$

Here, *s* represents the lateral shift in the focal spot from the optical axis, commonly referred to as the image height, and is a function of the incident angle *θ*. Common forms for this relationship used in optical design include *s* = *f*·tan(*θ*), *s*= *f*·sin(*θ*), and *s* = *f*·*θ*. In this work, we adopt *s* = *f*·*θ*. While this approximation, derived from the tangent relationship, does not yield perfect geometric imaging (introducing predictable distortion), it results in target phase curves for different angles that are more closely aligned in shape compared to the other two alternatives, thereby facilitating the numerical optimization process. The resulting distortion can be effectively corrected through post-processing.

The target phase profile derived from Equation (2) resembles a quadratic form. However, due to the refractive index mismatch at the substrate–air interface, Snell’s Law causes incident rays to bend toward the optical axis. This bending introduces angle-dependent deviations, so that the ideal target phase profiles diverge from each other; these deviations become especially pronounced at large incident angles. To mitigate these angle-dependent deviations and improve the overlap and smoothness of the phase profile across the field-of-view, making them more amenable for approximation, we employed a compensation term of the form *k*·sin(*δ_i_*) added to the phase calculated from Equation (2) [24], where *k* is the magnitude of wavevector in the air. For each discrete incident angle, a specific, small offset value *δ_i_* was empirically determined based on the specific system parameters. While the approach is empirical and not a complete physical model, applying these offsets effectively reduces the disparity between target profiles for different angles and provides a more consistent baseline for subsequent optimization fitting, as conceptually illustrated in Figure 1d.

As highlighted, achieving wide-angle imaging requires angle-dependent phase profiles. Nevertheless, a single-layer metasurface can only implement a fixed, angle-independent phase profile at each spatial location. Therefore, the core design challenge is to find the phase profile that best approximates the set of angle-dependent target profiles over the desired field-of-view (range of *θ*) and metasurface aperture (range of *r*). To address this, we employed a numerical optimization approach, which we term Gradient-Optimized Phase Profile Shaping (GOPP). This method aims to determine the optimal single, angle-independent phase profile. In the GOPP approach, the single, angle-independent phase profile $\hat{\phi }(r)$ was parameterized using an even-order polynomial function as expressed by Equation (3) [22,35]:

(3) $$\hat{\phi }(r)=\sum_{n=1}^{N}a_{n}{\left(\frac{r}{R}\right)}^{2n},$$

The polynomial coefficients *a_n_* served as the optimization variables. Here, *n* represents the even order of the polynomial, *r* denotes the radial coordinate on the metasurface, and *R* is the normalized radius. This function is appropriate given the shape of the target profile while providing both symmetry compatibility and low model complexity. In our optical setup, an aperture was introduced before the metasurface, leading to angularly dependent illumination of different, partially overlapping regions of the metasurface. As a result, individual metasurface coordinates are associated with multiple target phase values corresponding to different incident angles. The goal of the optimization was to minimize the mean squared error (MSE) between the polynomial-fitted phase $\hat{\phi }$ and the target phase *ϕ_t_* over both the relevant spatial coordinates of the metasurface and the range of incident angles:

(4) $$Loss=\frac{1}{N}\sum_{m=1}^{M}\sum_{l=1}^{L_{m}}{\left(\phi_{t}(\theta_{m},r_{l})-\hat{\phi }(r_{l})\right)}^{2},$$

Specifically, we discretized the angular range from 0° to 45° into *M* angles, denoted as *θ*_1_, *θ*_2_, …, *θ_M_*. For each incident angle, phase targets were sampled at *L* discrete positions across the metasurface. However, due to the front aperture and angle-dependent projection of incident rays, only a subset *L_m_* of these coordinates was illuminated under angle *θ_m_*. Consequently, the total number *N* of valid coordinate–angle combinations used in the loss function is given by the following:

(5) $$N=\sum_{m=1}^{M}L_{m}$$

Minimization was performed using a gradient-based optimizer, specifically the Adaptive Moment Estimation (ADAM) algorithm, which iteratively adjusted the polynomial coefficients to minimize the defined loss function. The optimization converged after about 50 epochs, each consisting of 500 iterations, with a total runtime of approximately 520 s on an NVIDIA RTX 4090 GPU.

Based on the methodology above, we designed a single-layer metalens system for operation at a wavelength of 803 nm with a period of 480 nm. The specific parameters are illustrated in Figure 1e, detailing a system incorporating a 2.4 mm diameter metasurface positioned on a 0.7 mm thick substrate, with a 0.5 mm diameter front aperture placed in front of the metasurface, separated by a 0.55 mm air gap. To quantitatively evaluate the accuracy of the polynomial approximation and the effectiveness of the optimization, we conducted a detailed error analysis based on three key metrics, absolute phase error, modulo-2*π* phase error, and log-relative phase error percentage, whose logarithmic form ensures the validity and reliability of the data. Figure 1f summarizes the average errors over different incident angles, while Figure 1g presents the corresponding averages along the spatial coordinates of the metasurface. The results show that the absolute phase error generally increases with the field angle, indicating that phase fitting becomes more challenging at larger field angles. However, when phase periodicity is taken into account, the modulo-2*π* error remains relatively lower, reflecting improved phase fitting accuracy and minimal abrupt phase jumps exceeding 2*π*, an important factor in ensuring physical realizability of metasurfaces. In terms of log-relative error, the fitting accuracy is generally high across all angles and positions. A localized amplification is observed near the central region of the phase profile, where the target phases approach zero and small absolute deviations become more pronounced in relative terms. Nonetheless, this effect is spatially limited and does not compromise overall fitting quality. These findings support the effectiveness of this gradient-descent-based fitting strategy for the optimization task performed.

**Figure 2:**
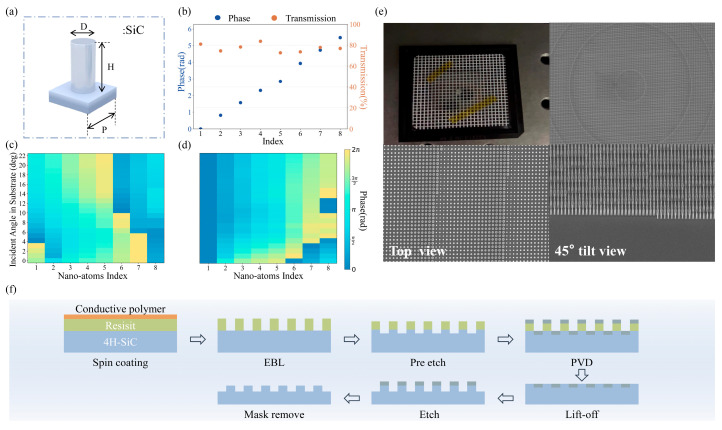
Metasurface design and fabricated structure. (**a**) Schematic of cylindrical SiC nano-atoms. (**b**) Transmission and phase responses for selected eight meta-atoms with different radius. (**c**) Phase responses for different meta-atoms under varying incident angles. (**d**) Relative phase variation, showing relative phase changes after subtracting the first meta-atom’s phase at each incident angle. (**e**) Fabricated metasurface sample, photograph, and top, 45° tilted view SEM image of the sample. (**f**) An illustration of the manufacturing process of the metalens.

To implement the design, we selected isotropic cylindrical nanopillars as the fundamental building elements (meta-atoms), as shown in Figure 2a.

This geometry was chosen for its inherent rotational symmetry, which offers a uniform optical response and simplifies fabrication. For the meta-atom material, we chose silicon carbide (SiC) for the high refractive index and minimal absorption losses in the target near-infrared range. We simulated the optical response of SiC nanopillar arrays with varying heights and radii using the Finite-Difference Time-Domain (FDTD) method to determine the optimal structural parameters. Based on criteria including relatively high transmission efficiency, full 2*π* phase coverage within a single layer, and compatibility with standard nanofabrication processes, eight distinct SiC nanopillar structures with a fixed height of 650 nm and radii ranging from 80 nm to 194 nm were chosen to discretize the continuous phase map. These selected SiC nanopillars exhibit transmission efficiencies exceeding 70% at the operating wavelength and collectively provide uniform 0–2π phase coverage essential for wavefront shaping (Figure 2b). Furthermore, a critical aspect for wide-angle performance is the behavior of these meta-atoms under oblique incidence. We analyzed the phase response variation in the selected unit cells across different incident angles within the operational FOV (Figure 2c,d). While the absolute phase value provided by each meta-atom naturally changes with the incident angle, we found that the relative phase trends among the set of eight cells are largely preserved at any given angle. This stability in relative phase ensures that the designed phase modulation pattern (which relies on the phase differences between meta-atoms) remains effective across the wide range of operating angles.

The metasurface was fabricated on a 4H-SiC substrate using standard semiconductor fabrication techniques. The detailed fabrication process flow [36], conceptually illustrated in Figure 2f, involved several key steps to transfer the designed pattern onto the SiC substrate. First, the SiC wafer was prepared by spin-coating an electron beam resist, followed by the deposition of a conductive layer to prevent detrimental charging effects. The high-resolution pattern of the metasurface was then defined using electron beam lithography (EBL), with potential double exposure employed to achieve precise pattern definition. After exposure, a suitable developer was used to selectively remove the unexposed resist. A brief pre-etch step was performed prior to metal deposition to ensure improved adhesion and quality of the subsequent mask layer. Next, a thin layer of Cr was deposited onto the sample via physical vapor deposition (PVD) to serve as a robust hard mask. A standard lift-off process was performed to remove the remaining resist and the overlying Cr, leaving the patterned Cr mask on the desired areas of the SiC surface. Finally, the defined Cr mask was used as a template to transfer the pattern into the SiC substrate using Reactive Ion Etching (RIE) to achieve the desired high aspect ratio and near-vertical sidewalls of the nanopillars. Figure 2e presents representative SEM images of the sample, including detailed top-down and 45° tilted views. These images visually confirm the excellent pattern fidelity, uniformity, and structural integrity of the fabricated SiC nanopillars across the metasurface.

## 3. Results and Discussion

**Figure 3:**
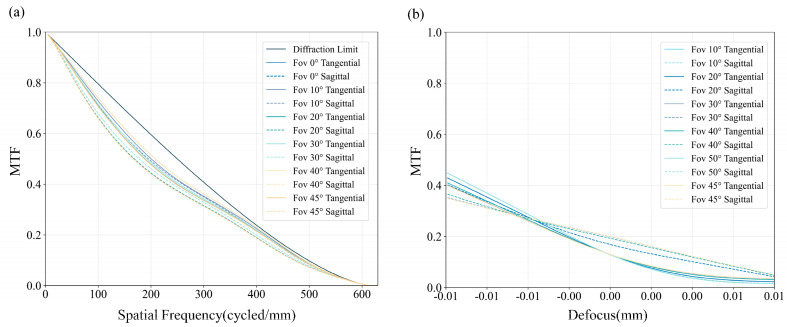
Simulations of the metasurface. (**a**) MTF curves of the metasurface at field angles (0° to 45°) in both tangential and sagittal planes. (**b**) Defocused MTF curves at the designed image plane.

The performance of the metasurface designed using the GOPP strategy was evaluated through optical simulations in Zemax OpticStudio. The optimized phase profile was implemented using a Binary 2 surface, with the fitted polynomial coefficients to model the designed phase distribution. Simulations of the modulation transfer function (MTF) were conducted across field angles from 0° to 45°, computed using Zemax’s built-in Fast Fourier Transform (FFT) diffraction algorithm, enabling an evaluation of image resolution and aberration control under ideal conditions. Both in-focus and slightly defocused MTF results are shown in Figure 3.

The results demonstrate stable and consistent MTF responses across the entire 90° FOV, confirming the design’s capability to deliver uniform image quality. The presented MTF curves correspond to the best-focus plane, defined by the minimum RMS wavefront error. While the system was designed with a nominal focal length of 1.000 mm, the simulated effective focal length (EFL) is slightly shorter at 0.998 mm, and the simulated best-focus plane also exhibits an axial shift, located at approximately 0.982 mm. This small axial deviation of the best-focus plane from the nominal design position is a common characteristic in wide-angle optical systems due to residual aberrations, and it does not fundamentally impact the overall focusing power or resolution. Given that practical systems often operate with a fixed image sensor plane, we further analyzed the axial sensitivity of image quality by computing defocus MTF curves at the nominal image plane of 1.000 mm, with results depicted in Figure 3b. We focused on a spatial frequency of 200 cycles/mm under defocus displacements of ±10 μm. The results reveal an asymmetric defocus response, with image quality degrading more rapidly under positive defocus compared to negative displacement. This behavior is commonly observed in systems with residual spherical aberration and indicates that the nominal image plane does not precisely coincide with the plane of best-focus for this design.

**Figure 4:**
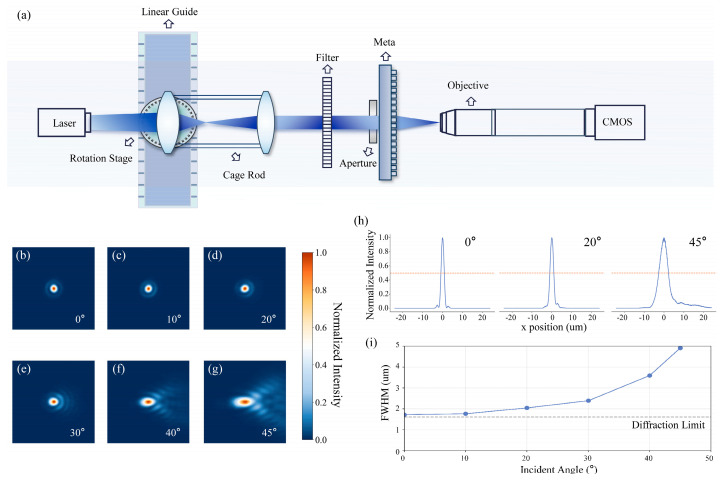
Experimental focusing results. (**a**) A schematic of the experimental setup, including a laser, linear guide, rotation stage, cage rod, color filter, metasurface, stop, objective, and CMOS camera. (**b**–**g**) Measured focus patterns when parallel light is incident at various angles (260 × 260 pixels). (**h**) Normalized intensity profiles along the *x*-axis (μm) for different incident angles. The dashed lines indicate the full width at half maximum (FWHM). (**i**) FWHM curve, as a function of the incident angle.

To experimentally verify the focusing capabilities of the fabricated metalens, an optical measurement setup was constructed as shown in Figure 4a. A collimated beam generated by a lens system was directed onto the metalens, which was mounted on a rotation stage to simulate different incident angles. A 20× microscope objective was used to better observe the focal spot captured by the CMOS.

Figure 4b–g displays the experimentally captured focal spot patterns at various field angles. To effectively present the critical information of the focal spot and remove uninformative background, the raw images were cropped to a central 260 × 260 pixel area, precisely centered on the brightest point, as the focused spot occupied only a small portion of the total sensor frame even under 20× magnification. At the optical axis (Figure 4b), the measured focal spot exhibits a FWHM of 1.716 μm, which is in close agreement with the theoretical diffraction limit of 1.606 μm, calculated based on the design wavelength and numerical aperture. This near-diffraction-limited performance confirms the high focusing quality of the metasurface under normal incidence. As the field angle increases, the focal spot undergoes significant degradation, transitioning from a symmetric spot on-axis to increasingly elongated and coma-like profiles at larger angles (Figure 4e–g). This evolution is further illustrated in the focal spot intensity cross-sections shown in Figure 4h, where the broadening and asymmetry become progressively more pronounced. As shown in Figure 4i, the corresponding FWHM increases gradually with the incident angle, remaining relatively low and nearly constant up to approximately 20°, but increasing more rapidly beyond 30°. This observed degradation is consistent with theoretical expectations regarding off-axis aberrations. Nevertheless, the focal spot remains discernible across all tested angles, indicating that the metasurface successfully maintains directional focusing despite the presence of these off-axis aberrations.

**Figure 5:**
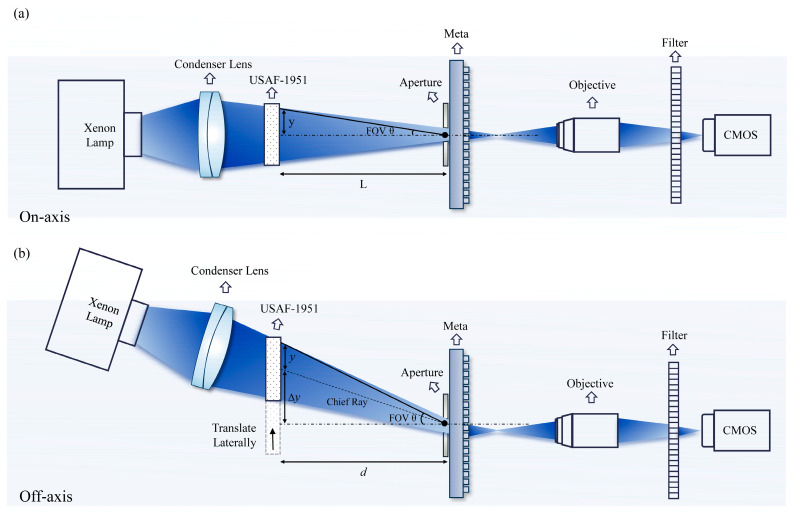
Imaging experimental setup. (**a**) A schematic of the imaging experimental setup on-axis, including a lamp, linear guide, color filter, metasurface, objective, and CMOS camera. (**b**) A schematic of the imaging experimental setup off-axis.

The imaging performance of the metalens was evaluated through an experiment using the setup shown in Figure 5.

A USAF 1951 resolution target was used as the object, placed at a distance of approximately 35 mm from the metalens. Illumination was provided by a broadband Xenon lamp and filtered by a bandpass filter (centered at 800 nm, 10 nm bandwidth) to match the design wavelength and minimize chromatic aberration. The intermediate image formed by the metalens was magnified by a 5× objective lens and captured by a CMOS camera. It is worth noting that, when placed 35 mm in front of the metalens with its center aligned to the optical axis, the diagonal extent of the object corresponds to a half-FOV of approximately 17°. This angle is calculated as tan^−1^(*y*/*d*), where *y* represents the half-extent of the object, and *d* is the object distance from the metasurface, as illustrated in Figure 5a.

To effectively extend the field-of-view beyond this limit imposed by the object physical size and illumination area, the object was progressively translated horizontally within the plane perpendicular to the optical axis. This systematic lateral movement, always in the same direction, gradually shifted the object’s center away from the lens center, which effectively increased the maximum off-axis angle between the object edge and the optical axis, thus expanding the effective field-of-view. Specifically, for a lateral shift in Δy, the effective maximum field angle is determined by tan^−1^((*y* + Δ*y*)/*d*), as further depicted in Figure 5b. To ensure proper illumination of the object across the entire scanning range, the positions and angles of the light source and condenser lens were appropriately adjusted during the movement. This method allowed peripheral object regions—those farthest from the lens center—to be effectively captured, thereby demonstrating an imaging capability spanning up to 45° half-FOV.

**Figure 6:**
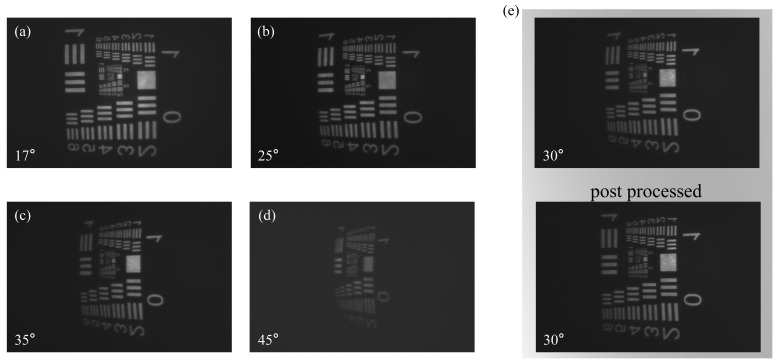
Imaging experiment results. (**a**–**d**) Experimental imaging results at various off-axis angles. (**e**) A comparison example of the actual captured image and the post-processed image at a 30° field-of-view.

Representative results are shown in Figure 6a–d. Due to the unidirectional lateral shift, the field distribution across each image is not radially symmetric. Instead, the maximum effective FOV typically appears near the left and lower edges of the captured frames for the respective off-axis positions.

Notable observations include reduced image brightness at larger angles and the presence of barrel distortion near the edge of the FOV. While simulations predict well-preserved imaging quality across the FOV, the experimental degradation at high angles is likely due to fabrication-related deviations and misalignment of the aperture, which are more impactful at large off-axis angles. To enhance the perceived image quality at the periphery affected by barrel distortion, a computational post-processing step involving a model-based distortion correction algorithm and unsharp mask sharpening (USM) was applied. As demonstrated in the example shown in Figure 6e, applying post-processing significantly mitigates the barrel distortion observed at a 30° angle, leading to a marked improvement in the perceived image quality and geometric fidelity. This demonstrates that computational post-processing can effectively compensate for geometric aberrations inherent in wide-angle metasurface imaging, enhancing the practical utility of the device.

## 4. Conclusions

In this work, we successfully demonstrated a compact, single-layer metasurface lens achieving wide field-of-view imaging with a total FOV up to 90°. This capability was realized through a gradient-based phase profile optimization strategy (GOPP). By incorporating a front aperture to better limit the range of rays, this system enables effective management of off-axis aberration within a planar structure. Experimental characterization validated the device’s performance, demonstrating consistent focusing and imaging capabilities across the designed angular range. While residual aberrations were observed at larger angles, computational post-processing was shown to effectively mitigate their impact on image quality. The resulting planar, miniaturized design offers inherent advantages for integration, presenting a promising route towards highly compact and broad angular imagers for applications like endoscopy and dermoscopy. Future work could explore extending the methodology to broadband operation, achieving varifocal capabilities via active elements, or integrating machine learning for adaptive aberration correction.

## Data Availability

The data underlying the results presented in this paper are not publicly available at this time but may be obtained from the authors upon reasonable request.
